# Factors Affecting Elevated Arsenic and Methyl Mercury Concentrations in Small Shield Lakes Surrounding Gold Mines near the Yellowknife, NT, (Canada) Region

**DOI:** 10.1371/journal.pone.0150960

**Published:** 2016-04-06

**Authors:** Adam James Houben, Rebecca D’Onofrio, Steven V Kokelj, Jules M Blais

**Affiliations:** 1 University of Ottawa—Program for Chemical and Environmental Toxicology, Department of Biology, University of Ottawa, Ottawa, ON, Canada, K1N 6N5; 2 NWT Geoscience Office, Government of the Northwest Territories, Yellowknife, NWT, Canada, X1A 2R3; National Institute of Technology Rourkela, INDIA

## Abstract

Gold mines in the Yellowknife, NT, region—in particular, the Giant Mine—operated from 1949–99, releasing 237,000 tonnes of waste arsenic trioxide (As_2_O_3_) dust, among other compounds, from gold ore extraction and roasting processes. For the first time, we show the geospatial distribution of roaster-derived emissions of several chemical species beyond the mine property on otherwise undisturbed taiga shield lakes within a 25 km radius of the mine, 11 years after its closing. Additionally, we demonstrate that underlying bedrock is not a significant source for the elevated concentrations in overlying surface waters. Aquatic arsenic (As) concentrations are well above guidelines for drinking water (10 μg/L) and protection for aquatic life (5 μg/L), ranging up to 136 μg/L in lakes within 4 km from the mine, to 2.0 μg/L in lakes 24 km away. High conversion ratios of methyl mercury were shown in lakes near the roaster stack as well, with MeHg concentrations reaching 44% of total mercury. The risk of elevated exposures by these metals is significant, as many lakes used for recreation and fishing near the City of Yellowknife are within this radius of elevated As and methyl Hg concentrations.

## Introduction

Resource extraction has the potential to result in severe contamination of surface waters. Giant Mine near Yellowknife, NT, is one of the world’s most prolific examples of local contamination from historical mining activities. Here, gold was found in ore bodies of arsenopyrite (FeAsS), where subsequent roasting was required, converting As and S to As_2_O_3_ and sulfur dioxide (SO_2_) before being vented to the atmosphere [1], along with other notable metals within the ore, such as antimony (Sb) [2]. During the roasting process, As is oxidized to arsenite and precipitated from the vapours as As_2_O_3_ [3,4] containing both As(III) and As(V) [5]. Most forms of As are toxic to organisms and inorganic arsenicals are carcinogenic [6,7]. The level of As toxicity is highly dependent on chemical speciation, where inorganic forms utilizing the trivalent As(III) ion are typically at the more toxic end of the scale [3,8]. Additionally, inorganic substances such as sulfide can complex strongly with As(III) species enhancing toxicity; while due to competitive binding, phosphate inhibits As adsorption to, or increases As leaching from mineral surfaces, enhancing inorganic As mobility [3,9]. Further, phosphate can also drive the biological processing of As-complexes [10] potentially leading to more toxic As compounds.

Beyond the mine property, the primary source of As pollution to landscapes is atmospheric deposition due to mining combustion processes being released from the roaster stacks. Estimates for roaster stack emissions of As to the atmosphere from 1947 to 1974, for Giant Mine and Con. Mine were 16,467 and 2,484 tons, respectively [1]. Atmospheric emissions consisted of both particulate and gaseous As, most likely as As_2_O_3_, though installation of scrubbers and filters reduced some of the particulate load over time. A wet scrubber was added in 1949. In 1951 and 1955 two electro-static precipitators were installed, and in 1958 a baghouse dust collector was added to reduce particulate emissions from the roaster stack, though increased roasting activity also occurred. These technologies reduced atmospheric emissions from 20,000 tons As_2_O_3_ per day to 454 kg/day, and then to 136–227 kg/day after the final baghouse installation [2]. Indeed, particulate removal was somewhat successful, though gaseous emissions continued.

Several studies of the landscape outside of the mine property have noted elevated concentrations of As and other related mining combustion by-products, such as higher sulfate and other trace metal concentrations in lake sediments [11,12] and soils and vegetation [2,13–15], as well as bird species [16]. These studies have attributed high As and S concentrations in both soils and vegetation up to 25 km away from the mine to emissions from the roaster stack [1,2]. The steep decline in As concentration with distance from source suggested relatively immediate atmospheric fallout as opposed to gaseous dispersion, likely due to rapid condensation of As_2_O_3_ vapour [1]. Wagemann et al. [17] noted that the surrounding soils and rock of the Yellowknife region, as a whole, are not abnormally high in arsenic, ranging from 2 to 10 ppm, though higher As concentrations do occur up to 140 ppm, mainly in pyrite and pyrrhotite. However, mine tailings at both Giant and Con. Mine are exceptionally high in As, ranging from 600–700 ppm [17]. Wagemann et al. (1978) was also one of the few studies to measure As in surface waters of lakes outside of these mine properties, though only five lakes were measured in total. Two of these lakes were directly connected to Con. Mine tailings ponds and had exceptional total As concentrations (up to 5.5 ppm), while three undisturbed lakes 10–26 km away from the mines were all lower than 0.01 ppm.

Hg is also often a by-product of smelters, and released to the atmosphere followed by wet/dry deposition to nearby catchment soils and water-ways, though due to the longer atmospheric residence time of Hg(0) we do not expect total Hg to demonstrate a local response within our study range of 25 km from the roaster stack. However, elevated sulfate associated with roasting at Giant Mine may enhance Hg methylation [18] in lakes closer to the smelters as any increase in sulfate concentrations up to 50 mg/L can stimulate sulfate reducing bacteria (SRB) activity [19]. Mercury is a naturally occurring metal and neurotoxin that is readily bioaccumulated by organisms once in the organic methylated form. Understanding Hg sources, methylation (and demethylation) processes, and where elevated Hg methylation may be occurring is important to environmental management and human health. To the best of our knowledge, elevated methyl Hg has not been reported in lakes or catchments around the Yellowknife mines, and has not been typically reported as a problem near mining roaster stacks.

Most Giant Mine pollution studies have detailed elevated As and other metal concentrations within the directly disturbed tailings ponds and in mine ground water interacting with underground stopes and chambers containing stored As. Of the studies measuring mining pollution relating to atmospheric deposition around the Giant Mine, most have focused on terrestrial soils and vegetation. Within the Yellowknife region, there have been few regional studies on the numerous small, shallow lakes exposed to atmospheric deposition from the mining and roasting processes of gold ore that occurred from 1949–1999 at the Giant Mine and to a lesser extent at the nearby Consolidated Mining and Smelting Company of Canada Ltd. (Con. Mine). This study investigates the extent of As contamination in freshwater lakes outside the mine boundaries, which are not connected to any downstream flow from tailings ponds or As waste storage-related groundwater. We also examine methyl Hg concentrations in lakes near Yellowknife to assess whether the spatial extent of mining-related atmospheric deposition influences aquatic Hg dynamics in local lakes. Specifically, we report water chemistry for 25 small lakes within a 25 km radius of the Giant Mine roaster stack to evaluate potential relationships between emission products (As, Sb, S) and related chemical constituents as a function of distance to the stack. Giant Mine was chosen as the study centre due to its long-term stack emissions, while Con. Mine was a smaller operation, producing less emissions, and ceased smelting altogether by 1971 [1]. Due to the relatively rapid atmospheric deposition of emitted roaster stack combustion by-products, we hypothesize elevated concentrations of arsenic, sulfate, and other related compounds in lakes closer to the stack and an exponential decline in concentrations with increasing distance from Giant Mine. We also hypothesize elevated methyl Hg is associated with elevated sulphate concentration proximal to the roaster stack. In addition, we hypothesize that the underlying bedrock (in particular the volcanic, arsenopyrite-rich bedrock) will not be a significant factor for elevated As, and other related metals.

## Methods

### Study Area

**Fig 1:**
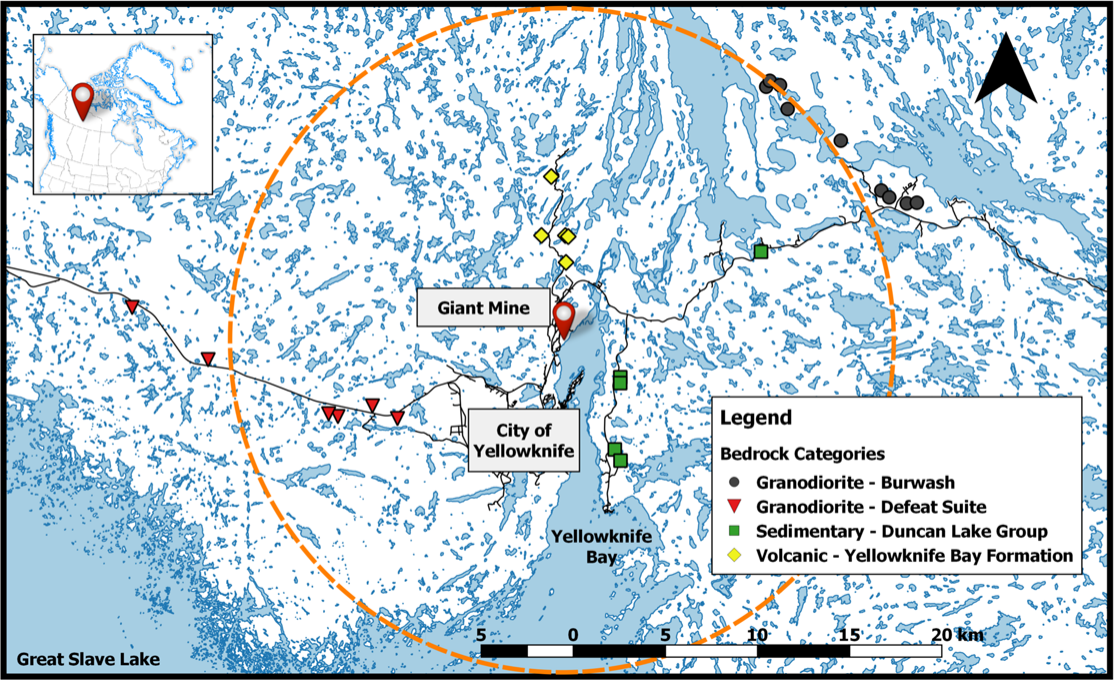
Location of 25 study lakes in the Yellowknife, NT, region categorized by underlying bedrock, centred on the Giant Mine roaster (red pin). The dashed ring indicates a 17 km radius from the roaster stack, a surface area of 921 km^2^, demarcating the regressed distance from the roaster for lakes that are above the CWQG for protection of aquatic life from As, set at 5 μg/L (CCME 1997). Inset indicates the location of Yellowknife, Northwest Territories, Canada, along Yellowknife Bay of Great Slave Lake. Map produced in QGIS using Canvec+, NRCan.

The 25 study lakes (Table 1) are small (0.4–17.8 ha; median of 2.9 ha), shallow (less than 4.3 m, with one exception at 11.5 m; median of 1.2 m) lakes within a 25 km radius of the Giant Mine roaster stack, which is located 5 km north of the city of Yellowknife, NT, (Fig 1). The gold ore zones mined are within the volcanic Yellowknife Bay Formation (YBF) within the Kam Group [20]. This volcanic formation is made up of mafic and felsic volcanic rocks, including basalts and andesites [21] containing high proportions of sulfides, including pyrite, arsenopyrite, sphalerite, chalcopyrite, stibnite, and Sb-bearing sulfosalts [22]. These minerals are the likely sources for the oxidized species of arsenic, antimony, and sulfur dust collected within roaster stack precipitators and baghouse filters, as well as the source for similar atmospheric particulate and gaseous emissions [23]. Five of the study lakes have catchments within this belt of volcanic bedrock. Metasedimentary rocks underlie study lakes along Detah Road to the southeast of the mine, while widespread intrusions of younger granodiorite underlie the catchments of many study lakes to the east (Burwash set) and west (Defeat Suite) of Yellowknife [21]. All 25 lakes are within the Level III Taiga Shield High Boreal (HB) ecoregion, with 14 lakes in the Level IV Great Slave Lowland HB. The remaining 11 lakes are in the Great Slave Upland HB [24], where much of the landscape has exposed outcrops of the Slave structural province of the Canadian Shield. The upland systems are generally bedrock dominated landscapes, with black spruce and jack pine forests growing in rock fractures and on discontinuous till and lacustrine deposits between bedrock exposures. The lowland systems are low-elevation granitic bedrock plains with discontinuous till and lacustrine deposits from glacial Lake McConnell between outcrops. Abundant bedrock and presence of permafrost in the unconsolidated sediments limit groundwater contributions and interaction between surface water and deeper, underlying soils. Mixed conifer and conifer-deciduous forests on and between rock outcrops are the main treed areas, while shore and floating fens and peat plateaus in wet depressions are present throughout. Prevailing winds are from the east, though during summer predominant wind energy is from the south [13,25,26].

**Table 1:** Physical characteristics of the 25 study lakes (2010) ordered by distance to the Giant Mine roaster stack, Yellowknife, NT, Canada. *dd* is decimal degree units. *D-Stack* is the lake distance to the mine roaster stack. *Z*_*max*_ is maximum lake depth.

Lake	Latitude (dd)	Longitude (dd)	Elevation (m)	D-Stack (Km)	Eco-Type	Bedrock	Secondary Class	Z_max_ (m)	Catchment Area (ha)	Lake Area (ha)	Catchment: Lake Ratio
01	62.530297	-114.351650	192	2.5	Lowland	Volcanic	Yellowknife Bay Fm	1.00	12.93	0.89	14.53
02	62.543164	-114.352419	193	3.9	Lowland	Volcanic	Yellowknife Bay Fm	0.50	3.55	0.43	8.26
03	62.542683	-114.349253	194	3.9	Lowland	Volcanic	Yellowknife Bay Fm	1.30	1.55	0.64	2.42
04	62.543200	-114.377928	198	4.2	Lowland	Volcanic	Yellowknife Bay Fm	1.22	15.73	13.07	1.20
05	62.475245	-114.293533	191	4.5	Lowland	Sedimentary	Metaturbidites	0.86	17.07	1.55	11.01
06	62.472445	-114.293622	195	4.8	Lowland	Sedimentary	Metaturbidites	2.00	29.08	2.49	11.68
07	62.571433	-114.367528	204	7.1	Upland	Volcanic	Yellowknife Bay Fm	2.20	52.99	2.70	19.63
08	62.440636	-114.299393	166	7.9	Lowland	Sedimentary	Metaturbidites	1.15	21.21	5.56	3.81
09	62.435292	-114.293284	163	8.5	Lowland	Sedimentary	Metaturbidites	1.92	15.35	1.35	11.37
10	62.535330	-114.142851	171	10.9	Upland	Sedimentary	Metaturbidites	0.67	39.44	2.88	13.69
11	62.455219	-114.531467	194	11.2	Lowland	Granodiorite	Defeat Suite	4.30	15.05	1.03	14.61
12	62.461200	-114.558220	191	12.1	Lowland	Granodiorite	Defeat Suite	0.90	45.75	2.55	17.94
13	62.456072	-114.595051	186	14.0	Lowland	Granodiorite	Defeat Suite	1.15	47.44	3.82	12.42
14	62.457357	-114.604978	185	14.5	Lowland	Granodiorite	Defeat Suite	1.05	33.82	8.81	3.84
15	62.603725	-114.113978	169	16.0	Upland	Granodiorite	Burwash	4.30	32.18	2.67	12.05
16	62.614448	-114.136894	176	16.0	Upland	Granodiorite	Burwash	0.97	23.90	3.58	6.68
17	62.617269	-114.133060	176	16.4	Upland	Granodiorite	Burwash	3.50	136.62	2.98	45.85
18	62.615192	-114.122138	192	16.6	Upland	Granodiorite	Burwash	1.48	18.82	1.11	16.95
19	62.588500	-114.057050	173	17.3	Upland	Granodiorite	Burwash	1.10	41.69	8.52	4.89
20	62.564500	-114.013818	228	18.3	Upland	Granodiorite	Burwash	11.50	32.05	11.53	2.78
21	62.561215	-114.005324	227	18.5	Upland	Granodiorite	Burwash	2.10	40.44	17.83	2.27
22	62.558277	-113.986598	231	19.4	Upland	Granodiorite	Burwash	1.00	22.16	4.37	5.07
23	62.558581	-113.975771	234	19.9	Upland	Granodiorite	Burwash	1.05	29.67	5.55	5.35
24	62.482972	-114.734067	179	20.2	Lowland	Granodiorite	Defeat Suite	4.25	54.73	1.28	42.76
25	62.507736	-114.815690	179	24.2	Lowland	Granodiorite	Defeat Suite	1.20	19.66	3.50	5.62

### Field Sampling

The 25 study lakes were selected for nearby road access while still being removed from any direct anthropogenic influence, such as roads bisecting catchments. Study locations did not require specific permission, while the overall research permit was approved by the Aurora Research Institute, Inuvik, NT; application number 1427. Surface water grab samples (0.5m) were collected July 21–28, 2010, from lake-centre by boat, using gloves; 10% of lake samples for all parameters were performed in triplicate for quality control. Prior to sampling, bottles (polyethylene) and caps were cleaned with dilute HCl (3-d soak), followed by triple rinsing with distilled deionized water, (10% HNO_3_ was used for trace metal collection bottles). Bottles were triple rinsed with lake water and filled to the rim to void the sample of air. Samples for trace metal (e.g. As, Sb) analyses were preserved with concentrated HNO_3_ to 2% of sample volume (dissolved fractions preserved after filtration). Water samples were kept cool (4°C) and in the dark until analysis. Field temperature and specific (@ 25°C) conductivity (SpC) were determined with a YSI Model 85 multimeter.

### Water Analyses

Trace metals (EPA Method 208–1) were analyzed at Taiga Environmental Laboratory, Yellowknife, NT, a Canadian Association for Laboratory Accreditation Inc. (CALA) institute. Analyses were also performed at Environment Canada's National Laboratory for Environmental Testing (NLET) (CALA accredited), Burlington, ON, using Environment Canada standard operating procedures (SOP): nitrogen (N) (SOP#1170) phosphorus (P) (SOP#1190); pH was determined with a Thermo Orion Model 106 meter; Chloride (Cl^−^) and sulfate (SO_4_^2−^) major anions (SOP #1080); Calcium (Ca^2+^), magnesium (Mg^2+^), sodium (Na^+^) and potassium (K^+^) major cations (SOP #1061); Dissolved organic carbon (DOC) and dissolved inorganic carbon (DIC) (SOP #1021).

Mercury measurements were performed at the CALA accredited Laboratory for the Analysis of Natural and Synthetic Environmental Toxicants (LANSET), University of Ottawa. Total mercury (Hg_T_) concentrations were determined by cold-vapour atomic fluorescence (CVAF) spectrometry (DL = 0.2 ng L^−1^) as adapted to U.S. EPA Method 1631(E) using Tekran 2600 Analyzer manufacture suggestions, using fluorinated HDPE bottles (Fischer Scientific Cat. 03-312-15) sealed in plastic bags to prevent contamination [27,28] in lieu of glass. In brief, BrCl was added to samples to oxidize Hg to Hg^2+^ which was sequentially reduced with NH_2_OH•HCl to destroy free halogens. Hg was then completely reduced to volatile Hg^0^ with SnCl_2_; Hg^0^ was collected on a gold trap while purging the sample with N_2_ and then transported, following thermal desorption, to a second gold trap from which Hg^0^ was transported by an N_2_ stream to the CVAF spectrophotometer (Tekran Instrument Corp., Series 2600). Two Initial Precision Recovery (IPR) samples along with both field and lab blanks were analyzed prior to field samples. Ongoing Precision Recovery (OPR) samples were then analyzed after every ten field samples. Recovery (IPR + OPR) was 98.28% ± 0.72% based on concurrent analyses of NIST-certified stock solutions, with a r^2^ = 0.9996 for the calibration curve. Tekran 2600 analyzer (Tekran Inc.) detection limit was 0.1 ng/L. Instrument blanks averaged 0.01 ± 0.13 ng/L (1 standard deviation), while field blanks averaged 0.12 ± 0.17 ng/L.

MeHg_T_ was determined by capillary gas chromatography-atomic fluorescence spectrometry (GC-AFS), as per Cai et al. [29]. In brief, 1 L samples were passed through a sulfhydryl-cotton column. The column is then treated 6 times with a 1 mL mix of KBr/CuSO_4_ 1 M (2:1). The solution was extracted with 150 μL of methylene chloride under agitation for 30 minutes, and then centrifuged at 5000 rpm for 10 minutes. Final MeHg_T_ concentrations determined on an Ai Cambridge Model GC 94 Gas Chromatograph (800°C pyrolysis) equipped with a CTC A200S Autosampler and PSA Merlin Detector. The detection limit of the method was calculated as three times the standard deviation of baseline noise, which was 0.2 pg/L. After every five analyses of MeHg in lake samples, a blank sample was analyzed followed by a MeHg standard (2 pg/L) analysis for control.

### Statistical Treatment

Data were Ln-transformed prior to statistical analyses to meet assumptions of normality for parametric tests. A correlation matrix (Table 2) was created in order to determine key water chemistry parameters to test with the distance to the Giant Mine roaster stack. For the most affected and key chemical parameters, linear regression was then used to predict relevant distances to the roaster stack for toxicity thresholds. To test any confounding influence by the underlying bedrock, ANCOVA was used to compare lakes by bedrock, while controlling for distance to the roaster stack. Multiple linear regression using distance to the roaster stack and bedrock category as predictive variables was also used to test if either variable alone or in combination was the best predictor of analyte concentration in our study lakes. All statistical analyses were completed using SPSS 16.0 (2007).

**Table 2:** Pearson correlation coefficients for 25 study lakes, July 2010, located within a 25 km radius of the Giant Mine, Yellowknife, NT. Note, all variables were Ln-transformed. Bold type indicates p < 0.05

	D-Mine	CA:LA	Z_max_	pH	TN	TP	SO_4_	As_T_	Sb_T_	Al_T_	Fe_T_	Mn_T_	Hg_T_	MeHg	%MeHg
D-Mine	**1**	0.09	0.33	0.01	**-0.67**	**-0.62**	**-0.74**	**-0.77**	**-0.87**	0.36	0.06	-0.24	-0.19	**-0.52**	**-0.55**
CA:LA	0.09	**1**	0.16	**-0.48**	0.27	0.01	-0.27	-0.07	-0.05	**0.40**	**0.47**	-0.32	**0.42**	0.18	-0.08
Z_max_	0.33	0.16	**1**	-0.22	-0.53*	-0.52*	-0.18	-0.31	-0.22	-0.02	-0.23	-0.26	-0.08	-0.26	-0.28
pH	0.01	**-0.48**	-0.22	**1**	-0.16	0.03	0.29	0.15	0.14	-0.34	**-0.71**	-0.01	-0.19	0.02	0.12
TN	**-0.67**	0.27	-0.53*	-0.16	**1**	**0.65**	**0.43**	**0.53**	**0.62**	-0.06	0.33	0.28	**0.42**	**0.43**	0.33
TP	**-0.62**	0.01	-0.52*	0.03	**0.65**	**1**	**0.45**	**0.76**	**0.68**	0.06	0.18	**0.47**	0.36	**0.59**	**0.43**
SO_4_	**-0.74**	-0.27	-0.18	0.29	**0.43**	**0.45**	**1**	**0.57**	**0.71**	**-0.48**	**-0.42**	-0.01	0.00	0.33	**0.57**
As_T_	**-0.77**	-0.07	-0.31	0.15	**0.53**	**0.76**	**0.57**	**1**	**0.93**	-0.29	-0.11	0.26	0.10	**0.50**	**0.44**
Sb_T_	**-0.87**	-0.05	-0.22	0.14	**0.62**	**0.68**	**0.71**	**0.93**	**1**	-0.33	-0.14	0.16	0.17	**0.56**	**0.50**
Al_T_	0.36	**0.40**	-0.02	-0.34	-0.06	0.06	**-0.48**	-0.29	-0.33	**1**	**0.47**	-0.02	**0.52**	-0.09	-0.42
Fe_T_	0.06	**0.47**	-0.23	**-0.71**	0.33	0.18	**-0.42**	-0.11	-0.14	**0.47**	**1**	0.15	0.20	0.19	-0.07
Mn_T_	-0.24	-0.32	-0.26	-0.01	0.28	**0.47**	-0.01	0.26	0.16	-0.02	0.15	**1**	0.02	0.02	-0.14
Hg_T_	-0.19	**0.42**	-0.08	-0.19	**0.42**	0.36	0.00	0.10	0.17	0.52	0.20	0.02	**1**	0.38	-0.03
MeHg	**-0.52**	0.18	-0.26	0.02	**0.43**	**0.59**	0.33	**0.50**	**0.56**	-0.09	0.19	0.02	0.38	**1**	**0.74**
%MeHg	**-0.55**	-0.08	-0.28	0.12	0.33	**0.43**	**0.57**	**0.44**	**0.50**	-0.42	-0.07	-0.14	-0.03	**0.74**	**1**

*—significance due to one deep outlier lake; not significant once removed

## Results

**Fig 2:**
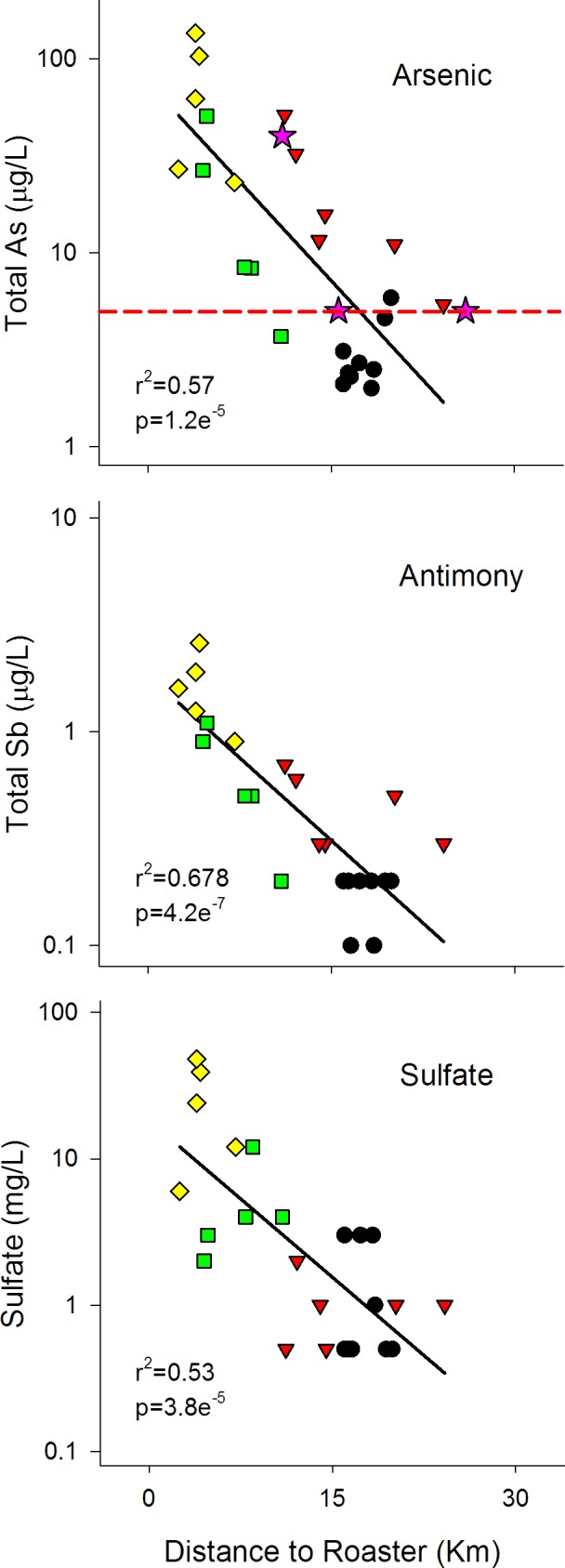
Ln-transformed lake water concentrations of total arsenic, total antimony, and sulfate in 25 lakes within the Yellowknife, NT, region. Lakes are plotted by underlying bedrock—Yellow diamond: Volcanic Yellowknife Bay Formation; green square: sedimentary Metaturbidites; red triangle: Granodiorite west; black circle: Granodiorite east; purple star: 3 undisturbed lakes from Wagemann et al 1978. The red dashed line indicates the 5 μg/L arsenic water quality guideline for the protection of aquatic life (CCME 1997), intersecting with the regression line at 17 km from the roaster.

We found the highest As and MeHg concentrations in lake water in sites closest to the Giant Mine roaster stack, strongly suggesting the influence of atmospheric deposition from the roaster on surface waters. Log-transformed total As concentrations demonstrated a significant negative relationship with distance to the Giant Mine roaster stack (Fig 2). These ranged from 27–136 μg/L at small lakes less than 4 km from the roaster stack to as low as 2.0 μg/L in lakes ranging 18–24 km from the roaster stack. When separated by underlying bedrock categories, average (standard deviation) As_T_ was 70.3 (48.9), 21.2 (17.4), 19.5 (19.5), and 3.1 (1.3), μg/L for YBF, western granodiorite sedimentary, and eastern granodiorite, respectively. Of these 25 study lakes, 16 were found to be above the 5 μg/L threshold for the protection of aquatic life (CCME 1997). Using the calculated regression, we estimate that within a radius of 17 km to the Giant Mine roaster stack, lakes are predicted to be above this 5 μg As/L threshold. Within this predicted 17 km radius “hot zone” from the Giant Mine roaster stack, only 5 lakes were below this threshold and were located as a cluster in the Burwash geological formation 16.0–16.6 km east of the roaster stack (Fig 1). Outside of this 17 km radius, several lakes found 20.2–24.2 km to the west of the roaster stack were above the threshold.

Total antimony (Sb_T_) concentrations ranged from 0.10–2.60 μg/L, with median of 0.30 μg/L, while sulfate was 0.50–48.00 μg/L, median of 2.00 μg/L. Both Sb_T_ and sulfate were negatively correlated to distance from the Giant Mine roaster stack (Fig 2), with lakes nearest to the stack having concentrations 10 times greater than the most distant lakes surveyed. Again, the lowest concentrations of both Sb_T_ and sulfate were measured in the most northeastern lakes located at distances greater than 16.0 km from the stack. Additionally, all three suspected mine-related water chemistry variables, (As, Sb, SO_4_), were highly correlated to each other (Table 2), in particular As and Sb (r = 0.93). To contrast, several metals (total Al, Fe, and Mn) had aquatic concentrations that were not significantly correlated with the distance to the roaster stack (p>0.05), while Al and Fe were both positively correlated to CA:LA ratios (Table 2). Although not reported in figures or summary table, Ca^2+^ ion concentrations were also found to be negatively correlated (r = -0.55, p = 0.005) to the distance from the roaster stack.

**Fig 3:**
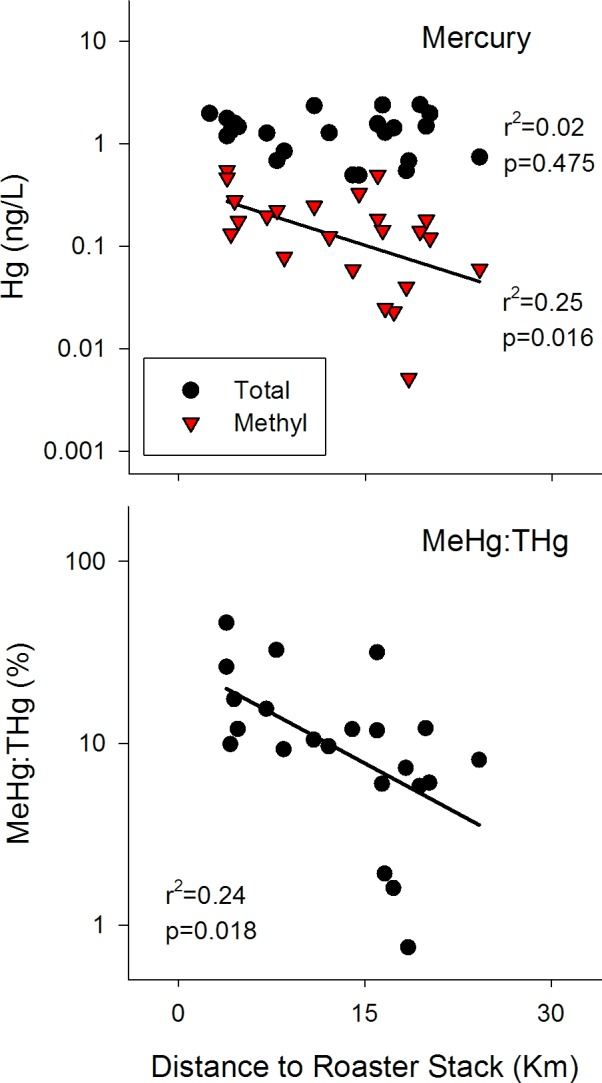
top: Ln-transformed lake water concentrations of total (black circle) and methyl mercury (red triangle); bottom: proportion of THg that is MeHg, in 25 lakes within the Yellowknife, NT, region.

Total mercury (Hg_T_) concentrations ranged from 0.49 to 2.41 ng/L; methyl mercury (MeHg) concentrations from 0.01 to 0.55 ng/L; resulting in MeHg:Hg_T_ ratios ranging from 1% to 46%; median of 10%. Most natural lakes correspond to a less than 1% MeHg conversion ratio [30]. Further, within a review of 6 studies surveying 74 lakes across Canada's Arctic, average %MeHg ranged from 2.9% to 8.0% [31]. Total Hg was unrelated to roaster stack distance (r = -0.19), but was positively correlated with catchment to lake area ratios (CA:LA) (r = 0.42) and DOC (r = 0.38) (Table 2). MeHg concentrations were indeed higher nearer to the roaster stack (Fig 3), though not correlated with CA:LA nor above the 4 ng MeHg/L guideline for the protection of aquatic life (CCME, 1987). The MeHg:Hg_T_ ratio was also negatively related to distance from the roaster stack (r = -0.55, Table 2; r^2^ = 0.24, Fig 3) and though significant, some of the remaining variation may be due to seasonal and annual variation of complex whole lake systems. Further, the MeHg:Hg_T_ ratio was positively related to sulfate (r = 0.57), though it was not correlated to DOC (r = 0.06) or CA:LA (r = 0.09) (Table 2).

**Fig 4:**
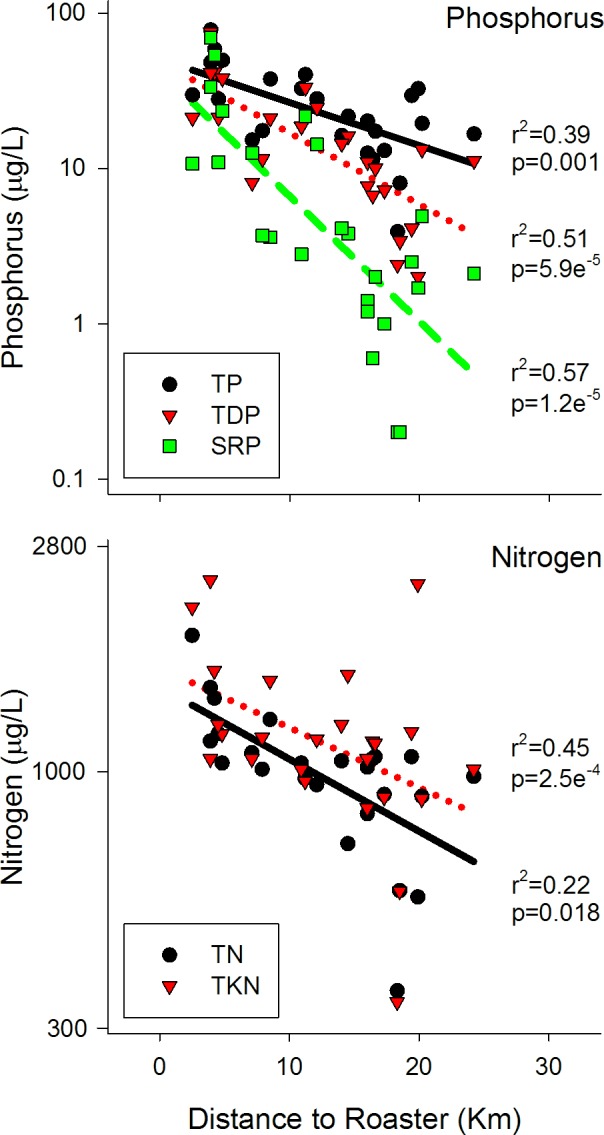
Aquatic phosphorus and nitrogen concentrations in study lakes as relating to distance to the Giant Mine roaster stack. n = 25. Total (TP), total dissolved (TDP), and soluble reactive (SRP) phosphorus, as well as total (TN), total Kjedhal (TKN) nitrogen concentrations were measured.

Minimum concentrations for total phosphorus (TP), total dissolved (TDP), and soluble reactive (SRP) were 3.90, 2.00, and 0.20 μg/L, while maxima were 77.4, 74.9, and 69.1 μg/L, with regression coefficients of r^2^ = 0.39, 0.51, and 0.57, respectively (Fig 4). Concentrations for total nitrogen (TN) ranged from 365–1870 μg/L and total Kjeldahl nitrogen (TKN) from 347–2410 μg/L, with r^2^ = 0.45 and 0.22, respectively (Fig 4). The proportion of TDP and SRP was inversely related to distance from Giant Mine, ranging from over 90% for both TDP and SRP in our nearest lakes less than 4 km from the roaster stack; to as low as 6% TDP and 5% SRP at our distant lakes more than 20 km from the stack. To note, N and P measurements were also inversely correlated to lake depth (Table 2), however this is likely due to one lake (lake 20) being nearly three-fold deeper than the next deepest lake in our study, and once lake 20 was removed any relationship between depth and total N and total P was not significant, except SRP. SRP was still found to have a significant inverse relationship with lake depth with or without lake 20 included. No other water chemistry variables, including As and MeHg, were observed to correlate with lake depth. Additionally, percent dissolved oxygen ranged from 47% to 135% (median of 94%, SD = 19%).

Controlling for distance to the roaster stack using ANCOVA, resulted in significant differences between underlying bedrock categories for each of As, Sb, and SO_4_ (S1 Table). However, the estimated marginal means, (the adjusted means after controlling for the covariate of distance), indicated that the western granodiorite lakes had the highest As concentrations, more than doubling the volcanic Yellowknife Bay Formation that contains arsenopyrite. For Sb, both the Yellowknife Bay Formation and western granodiorite categories were similar, having the highest estimated marginal mean concentrations. For sulfate, estimated marginal means suggested that the Yellowknife Bay Formation had by far the highest concentrations once distance was considered. Methyl Hg ANCOVA results demonstrated no significant relationship to bedrock categories, regardless of controlling for distance to the roaster stack (S1 Table).

Using both distance to the roaster stack and bedrock category as predictive variables in backward stepwise multiple linear regression, we observed a variable response from our dependent variables (As, Sb, SO_4_, and MeHg) to either predictor (S2 Table). Both As and Sb suggested that both distance and bedrock together produced the strongest models (adjusted r^2^ = 0.69 and 0.76, p = 1.1E-06 and 4.8E-08, respectively), though both models indicate that distance was the greater contributor via p-partial statistics. For sulfate, bedrock category was a marginally stronger predictor alone (adjusted r^2^ = 0.65, p = 7.0E-07) than it was when paired with distance (adjusted r^2^ = 0.65 p = 3.6E-06). Methyl Hg was most strongly predicted by distance alone (adjusted r^2^ = 0.27, p = 4.3E-03) over any model that also included bedrock category, whereas Hg_T_ demonstrated no significant relationship to either distance to roaster stack or underlying bedrock. The regression coefficient may also be affected by seasonal and inter-annual variation within each catchment.

## Discussion

Briefly, our analysis shows a clear point source distribution of arsenic in surface waters surrounding the gold mines near Yellowknife, NT, and that the concentrations of arsenic are well above drinking water quality guidelines (10 μg/L) and protection for aquatic life (5 μg/L) for lakes within 17 km of the Giant Mine roaster stack (Fig 2). This relationship is not surprising when considering that approximately two thirds of baghouse dust from the roaster stack is composed of As_2_O_3_ [23]. Antimony was also highly correlated with arsenic (r = 0.93, Table 2) and was also shown to be the third largest oxide concentration in the baghouse dust collections. Similar trends for both antimony and sulfur radiating from the roaster stack were also observed (Fig 2), indicating a depositional influence for these two elements on our lakes for at least the 25 km limit of our study set. As well, MeHg concentrations are higher nearer to the roaster stack (Fig 3) and MeHg/Hg_T_ ratios are exceptionally higher when contrasting with typical natural waters; neither have been reported in any prior analyses of Giant Mine pollution.

We demonstrate statistically that the relationship of aquatic As concentrations to the roaster stack is unlikely related to any influence from the underlying volcanic arsenopyrite-rich YBF bedrock, from which the gold is mined. ANCOVA analysis using distance to roaster stack as a covariate showed that the volcanic YBF bedrock is not associated with the highest aquatic As concentrations (S1 Table). Further, the western Granodiorite lakes demonstrated the highest estimated marginal means, even though they are 11–24 km West of the roaster stack, and not the lakes with catchments underlain by the arsenopyrite-rich volcanic rocks. The importance of distance to the stack was also reflected in the stepwise multiple regression as both bedrock category and distance to roaster stack are included in the optimal model (S2 Table). Though the YBF lakes have the highest average As_T_ concentrations as a bedrock category, the more distant western Granodiorite lakes likely demonstrate the highest estimated marginal means for As_T_ concentrations in our ANCOVA due to downwind effects from the predominantly easterly winds in the region [13,25,26], as opposed to underlying geology.

ANCOVA results for MeHg showed that bedrock category had no significant influence on MeHg concentrations (S1 Table), which was also reflected in multiple regression results where only distance to the mine was selected as the stronger model (S2 Table). Sulfate concentrations in lake water decreased significantly with distance to the roaster stack (Fig 2), however, both ANCOVA and multiple regression results suggested a potential bedrock source relating to the YBF. However, it is difficult to delineate if this is related to underlying bedrock influences or to more rapid atmospheric fall-out once emitted from the stack.

It is noteworthy that both Fe and Al total metal concentrations were not related to distance from the roaster stack (Table 2), as they are the second and fourth most dominant oxides in baghouse dust collections, respectively [23]. Sb was the third most abundant oxide in the baghouse collections and was significantly correlated to distance to roaster stack. All three of these metal oxides (Fe_3_O_3_, SbO, Al_2_O_3_) were less than 1.6% of the baghouse dust concentrations, while As_2_O_3_ was approximately 65%. This highlights that the Sb signal was observed due to its relatively low environmental background abundance, while any Al and Fe deposition was negligible to environmental background concentrations. Also, using environmentally abundant metals that were not present in baghouse dust collections (e.g. Mn), we further demonstrate that no discernible regional patterns are potentially responsible for any similar trends that were observed for our metals of concern: As, Sb, Hg. In several other studies, for comparison, atmospheric deposition of mining-related metals (e.g. Cu and Ni) into nearby lakes were measured 50 km downwind prior to emissions reductions, and still 20–30 km from smelters post-reductions [32].

Arsenic speciation is of concern as this will ultimately determine bioavailability and the degree of toxicity to organisms. Within our study lakes, arsenic speciation is expected to be predominantly in the oxidized inorganic As(V) form as gaseous and particulate As_2_O_3_ is emitted from the roaster stack [23]. The atmospheric residence time is expected to be relatively short, depositing on the landscape as condensate [1]. Due to these lakes also being relatively shallow and oxygen-rich (average max depth of 2.1 m, median DO of 91%), the As(V) will likely remain as the dominant inorganic arsenic ion in surface waters. However, phytoplankton activity may also drive toxic As speciation depending on nutrient concentrations. Using phosphorus as an index of trophic status, our study lakes range from oligotrophic to highly eutrophic (Fig 4), and suggest that greater proportions of As(V) may be reduced by phytoplankton to the more toxic As(III) in higher phosphorus lakes [8,10,33]. Further investigation into the As speciation and bioaccumulation in biota is required to resolve the potentially compounded toxic effects in eutrophic lakes closer to the roaster stack.

Waste ponds on the Giant Mine site have measured total As concentrations up to 8 mg/L in tailings ponds and surface waters to over 4000 mg/L in underground mine seepages [23], however few studies have looked beyond the mine’s boundaries for surface water pollution. Wagemann et al [17] looked at 5 larger lakes in 1972–75, finding two of the lakes 10 and 12 km south of the Giant Mine roaster stack with elevated As concentrations between 0.7–5.5 mg/L. These two lakes were located immediately adjacent to the Con. Mine at the south of Yellowknife receiving both sewage waters and As-containing seepages from Pud Lake; a tailings pond. A third lake 10 km from Giant Mine and 4 km from Con. Mine was not directly connected to Con. Mine, which had just recently ceased roasting operations in 1971, and had much lower As concentrations of 0.01–0.07 mg/L. The remaining two undisturbed lakes were more distant, 16 and 26 km northeast of the Giant Mine roaster stack, and were both <0.01 mg/L. Excluding the directly disturbed tailings pond-contaminated lakes, the results for the three undisturbed lakes from the early 1970s fit within the 95% prediction interval of our current As model (Fig 2), indicating the lasting legacy effects of the Giant Mine roaster stack emissions on local lake systems over 10 years after mining activity has ceased, or much longer if considering the effectiveness of scrubbers and precipitators installed between 1949–1955.

Greater MeHg concentrations in lakes nearer to the roaster stack may be an indirect response to the significant increase in sulfate emissions and subsequent deposition into the nearby catchments from roaster emissions. In our study lakes, the range of %Hg_T_ as MeHg is 1–46%, with a median of 10%, which is notably higher than the expected 1% MeHg in most natural lakes [30] or even the 2.9% to 8.0% MeHg range observed in several more geographically relevant Arctic studies in Canada [31]. To contrast, within a site along the St. Lawrence River, near a pulp mill in Cornwall, Ontario, direct inputs of Hg pollution led to %MeHg in sediment pore water as high as 64% [34]. As indicated by our Hg_T_ relationship with CA:LA (Table 2), overland catchment run-off of Hg can be responsible for up to 94% of total Hg inputs to adjacent wetlands and lakes [35,36], and due to the strong affinity of Hg to sulfhydryl groups on DOC [37] it is not surprising to see a similar correlation. However, the increase in %MeHg may be due to enhanced MeHg production within catchments nearer to the roaster stack (Fig 3). Hg emissions from gold mining activities are well known [38], however due to the very long atmospheric residence time (0.5–1.0 years) of Hg it is not surprising that a point-source influence of Hg_T_ is not observed within our 25 km radius from the roaster stack. This result suggests that within-catchment processing of available Hg to MeHg may be elevated in lakes nearer to the roaster stack and the significant correlation between sulfate and %MeHg (Table 2) may corroborate this process.

Sulfur is likely emitted from the roaster stack as SO_2_, before being converted to sulfuric acid within the atmosphere and lake waters. This increase in atmospheric sulfate loading may ultimately lead to enhanced Hg methylation by sulfate reducing bacteria [19,39]. Further, our maximum sulfate concentration is 48 mg/L, thus below a generally accepted threshold of 50 mg/L where sulfate can begin limiting Hg methylation [19]. Also, it is not uncommon for sulfate and MeHg concentrations to be uncorrelated during high methylation periods, as sulfate is being consumed during the process. We also note that experiments by Dageune et al [40] demonstrated that greater base cation concentrations, in particular Ca^2+^ and Mg^2+^, inhibit Hg(II) uptake and subsequent methylation. In our study we observed significantly higher Ca^2+^ concentrations nearer to the roaster stack, which would suggest greater cation inhibition of Hg(II) and thus lower MeHg concentrations, however we observed greater MeHg, further pointing to sulfate as an influence on MeHg formation.

These increases in both As compounds and organic methyl Hg in surface waters due to direct and indirect influences from Giant Mine emissions are cause for concern of bioaccumulation in aquatic food-webs. Additionally, due to the ephemeral drainage of many of these small lakes, it is possible that transfer of materials from the affected catchments could continue for several years after the mine ceased roaster stack operations. Our results and the related hydrology imply that these lakes are sinks, not only for water but also for materials deposited on the catchments, suggesting greater implications for aquatic ecosystems. Considering that roaster stack emission-controls were installed at Giant Mine before 1960, the legacy contamination we measured 55 years later demonstrates a high persistence of arsenic and other stack emission products in these lakes.

Drinking water for the town of Yellowknife is sourced from the Yellowknife River, less than 5 km from the roaster stack. However, as the relationship between contaminants and distance declines exponentially from the mine, concentrations for both contaminants of concern are likely lower in the river due to its large watershed of over 10,000 km^2^, and have been monitored by the City of Yellowknife. To contrast, however, there are still numerous small lakes even closer to the mine than our survey sampled for, with likely equal or higher concentrations of As. The elevated total As and MeHg concentrations in lake waters within approximately 20 km of the Giant Mine roaster stack, will potentially be of concern for enhanced bioaccumulation of these persistent pollutants in aquatic food webs.

## Supporting Information

S1 Table ANCOVA results for select water chemistry variables in Yellowknife lakes contrasting bedrock categories with distance to roaster stack as covariant. (DOCX)

S2 Table Multiple linear regression results of metals in lake water from the Giant Mine, Yellowknife, NT, region. (DOCX)

S3 Table Key water chemistry measurements for 25 small lakes within 25 km of the Giant Mine roaster stack, Yellowknife, NT, sampled July 21–28, 2010. (DOCX)
