# Supplementary material for: Factors Affecting Elevated Arsenic and Methyl Mercury Concentrations in Small Shield Lakes Surrounding Gold Mines near the Yellowknife, NT, (Canada) Region
Source: PLoS One. 2016 Apr 6;11(4):e0150960. doi: 10.1371/journal.pone.0150960 (PMC4822959; doi:10.1371/journal.pone.0150960)
Supplement: S1 Table — (DOCX) [file pone.0150960.s001.docx]

| Table S1: ANCOVA results for select water chemistry variables in Yellowknife lakes contrasting bedrock categories with distance to roaster stack as covariant. Bolded p-values indicate significant p<0.01. Bedrock categories: YBF=Yellowknife Bay Formation; SED=sedimentary metaturbidite; Gran=granodiorite east (E) and west (W) | | | | | | | |
| --- | --- | --- | --- | --- | --- | --- | --- |
| Dependent Variable | Bedrock summary statistics | | Estimated marginal mean concentration, calculated at distance = 2.4 km, grouped by bedrock category | | | | |
|  | F | p | YBF | SED | GranE | GranW | Units |
| As | 9.39 | **4.4E-04** | 12.18 | 6.55 | 6.49 | 29.67 | ug/L |
| Sb | 6.92 | **2.2E-03** | 0.63 | 0.37 | 0.28 | 0.61 | ug/L |
| SO4 | 6.06 | **4.2E-03** | 34.22 | 5.20 | 0.74 | 0.72 | ug/L |
| MeHg | 0.19 | 9.0E-01 | 0.15 | 0.13 | 0.10 | 0.16 | ng/L |
| THg | 0.46 | 4.6E-01 | 1.25 | 1.17 | 1.47 | 0.97 | ng/L |
