# Supplementary material for: Factors Affecting Elevated Arsenic and Methyl Mercury Concentrations in Small Shield Lakes Surrounding Gold Mines near the Yellowknife, NT, (Canada) Region
Source: PLoS One. 2016 Apr 6;11(4):e0150960. doi: 10.1371/journal.pone.0150960 (PMC4822959; doi:10.1371/journal.pone.0150960)
Supplement: S2 Table — (DOCX) [file pone.0150960.s002.docx]

| Table S2: Multiple linear regression results of several metals in lake water from the Giant Mine, Yellowknife, NT, region, and the independent variables Bedrock Category and Distance to Roaster Stack (Ln transformed). | | | | | | | |
| --- | --- | --- | --- | --- | --- | --- | --- |
| Dependent Variable | Adj. r^2^ | SE_EST_ | F | p | Predictor variables | Coefficient ± SE | p_(partial)_ |
| Ln As | 0.69 | 0.74 | 27.4 | 1.1E-06^a^ | Distance | -2.6 ± 0.4 | 2.4E-06^a^ |
|  |  |  |  |  | Bedrock | 0.7 ± 0.2 | 6.6E-03^a^ |
|  |  |  |  |  | Constant | 6.4 ± 0.6 | 1.6E-10^a^ |
| Ln Sb | 0.76 | 0.44 | 39.9 | 4.8E-08^a^ | Distance | -1.6 ± 0.2 | 1.7E-06^a^ |
|  |  |  |  |  | Bedrock | 0.3 ± 0.1 | 8.2E-02 |
|  |  |  |  |  | Constant | 2.1 ± 0.3 | 3.7E-06^a^ |
| Ln SO_4_^2-^ | 0.65 | 0.85 | 45.5 | 7.0E-07^a^ | Distance | -^b^ | -^b^ |
|  |  |  |  |  | Bedrock | -1.1 ± 0.2 | 7.0E-07^a^ |
|  |  |  |  |  | Constant | 3.7 ± 0.5 | 3.3E-08^a^ |
| Ln MeHg | 0.27 | 0.94 | 10.0 | 4.3E-03^a^ | Distance | -0.9 ± 0.3 | 4.3E-03^a^ |
|  |  |  |  |  | Bedrock | -^b^ | -^b^ |
|  |  |  |  |  | Constant | 0.1 ± 0.7 | 8.4E-01 |
| Ln THg | 0.00 | 0.48 | - | - | Distance | -^b^ | -^b^ |
|  |  |  |  |  | Bedrock | -^b^ | -^b^ |
|  |  |  |  |  | Constant | 0.2 ± 0.1 | 4.2E-02^a^ |
| ^a^ statistically significant (p<0.05) | | | | | | | |
| ^b^ removed in stepwise regression | | | | | | | |
