# Supplementary material for: Factors Affecting Elevated Arsenic and Methyl Mercury Concentrations in Small Shield Lakes Surrounding Gold Mines near the Yellowknife, NT, (Canada) Region
Source: PLoS One. 2016 Apr 6;11(4):e0150960. doi: 10.1371/journal.pone.0150960 (PMC4822959; doi:10.1371/journal.pone.0150960)
Supplement: S3 Table — (DOCX) [file pone.0150960.s003.docx]

| Table S3: Key water chemistry measurements for 25 small lakes within 25 km of the Giant Mine roaster stack, Yellowknife, NT, sampled July 21-28, 2010. Unless specified, concentrations are for total analyte analysis. (SpC: specific conductivity) | | | | | | | | | | | | | | | | | | | |
| --- | --- | --- | --- | --- | --- | --- | --- | --- | --- | --- | --- | --- | --- | --- | --- | --- | --- | --- | --- |
| Lake | pH | DIC | DOC | N | P | SpC | SO4 | Ca | Al | As | Fe | Li | Mn | Sb | Sr | U | Hg | Methyl Hg |  |
| # | - | mg/L | mg/L | µg/L | µg/L | µS/cm | mg/L | mg/L | µg/L | µg/L | µg/L | µg/L | µg/L | µg/L | µg/L | µg/L | ng/L | ng/L |  |
| 1 | 7.25 | 14.9 | 29.8 | 1870 | 29.6 | 137 | 6.0 | 24.4 | 24.0 | 26.9 | 338.0 | 3.5 | 37.6 | 1.6 | 50.4 | 0.1 | 1.98 |  |  |
| 2 | 8.51 | 31.0 | 25.2 | 1470 | 77.4 | 376 | 48.0 | 45.8 | 16.0 | 136.0 | 20.0 | 9.6 | 7.6 | 1.9 | 162.0 | 0.6 | 1.20 | 0.55 |  |
| 3 | 7.51 | 35.4 | 23.2 | 1150 | 47.9 | 343 | 24.0 | 47.9 | 6.0 | 62.4 | 46.0 | 6.8 | 16.1 | 1.3 | 118.0 | 0.3 | 1.77 | 0.47 |  |
| 4 | 8.85 | 18.8 | 25.2 | 1400 | 58.2 | 265 | 39.0 | 30.1 | 28.0 | 103.0 | 22.0 | 3.8 | 82.7 | 2.6 | 136.0 | 0.2 | 1.33 | 0.13 |  |
| 5 | 7.36 | 9.9 | 33.4 | 1190 | 27.8 | 105 | 2.0 | 11.2 | 39.0 | 26.6 | 574.0 | 5.1 | 12.9 | 0.9 | 50.2 | 0.1 | 1.59 | 0.28 |  |
| 6 | 6.87 | 7.1 | 30.4 | 1040 | 49.5 | 75 | 3.0 | 7.7 | 159.0 | 50.7 | 594.0 | 3.8 | 29.7 | 1.1 | 32.0 | 0.1 | 1.47 | 0.18 |  |
| 7 | 8.54 | 19.4 | 22.0 | 1090 | 15.2 | 192 | 12.0 | 33.0 | 6.0 | 23.1 | 10.0 | 1.6 | 9.5 | 0.9 | 36.4 | 0.1 | 1.27 | 0.20 |  |
| 8 | 8.00 | 16.8 | 27.8 | 1010 | 17.4 | 195 | 4.0 | 17.2 | 8.0 | 8.4 | 311.0 | 7.4 | 15.0 | 0.5 | 86.0 | 0.1 | 0.69 | 0.22 |  |
| 9 | 7.45 | 18.4 | 33.5 | 1270 | 37.5 | 205 | 12.0 | 20.4 | 14.0 | 8.3 | 192.0 | 11.5 | 33.1 | 0.5 | 81.6 | 0.1 | 0.85 | 0.08 |  |
| 10 | 8.01 | 5.1 | 30.6 | 1040 | 32.6 | 73 | 4.0 | 6.5 | 169.0 | 3.7 | 550.0 | 4.8 | 12.3 | 0.2 | 27.7 | 0.2 | 2.35 | 0.25 |  |
| 11 | 7.29 | 4.5 | 34.8 | 969 | 40.0 | 62 | 0.5 | 6.9 | 35.0 | 51.4 | 308.0 | 4.2 | 38.3 | 0.7 | 22.2 | 0.1 |  |  |  |
| 12 | 7.41 | 5.9 | 29.9 | 941 | 28.0 | 71 | 2.0 | 8.5 | 97.0 | 32.3 | 287.0 | 3.6 | 13.3 | 0.6 | 32.7 | 0.1 | 1.28 | 0.12 |  |
| 13 | 7.67 | 16.7 | 25.8 | 1050 | 16.2 | 259 | 1.0 | 22.7 | 19.0 | 11.6 | 361.0 | 4.5 | 31.6 | 0.3 | 80.3 | 0.1 | 0.49 | 0.06 |  |
| 14 | 8.08 | 21.8 | 30.0 | 718 | 21.6 | 298 | 0.5 | 23.9 | 10.0 | 15.7 | 301.0 | 7.3 | 33.4 | 0.3 | 100.0 | 0.1 | 0.49 | 0.33 |  |
| 15 | 7.56 | 8.0 | 31.4 | 1020 | 12.5 | 87 | 0.5 | 9.9 | 88.0 | 3.1 | 308.0 | 5.4 | 7.8 | 0.2 | 46.5 | 1.2 | 1.57 | 0.19 |  |
| 16 | 7.04 | 12.4 | 22.8 | 824 | 20.1 | 131 | 3.0 | 13.8 | 52.0 | 2.1 | 374.0 | 5.5 | 16.0 | 0.2 | 71.8 | 0.8 | 1.56 | 0.49 |  |
| 17 | 7.64 | 7.4 | 31.2 | 1060 | 11.4 | 82 | 0.5 | 9.2 | 60.0 | 2.4 | 150.0 | 5.9 | 7.3 | 0.2 | 38.7 | 1.2 | 2.39 | 0.14 |  |
| 18 | 6.41 | 6.2 | 31.2 | 1070 | 17.3 | 71 | 0.5 | 7.1 | 99.0 | 2.3 | 793.0 | 4.6 | 31.5 | 0.1 | 33.1 | 0.2 | 1.29 | 0.02 |  |
| 19 | 7.90 | 8.5 | 23.0 | 900 | 13.0 | 91 | 3.0 | 10.0 | 38.0 | 2.7 | 153.0 | 7.6 | 11.0 | 0.2 | 47.2 | 1.7 | 1.43 | 0.02 |  |
| 20 | 7.94 | 6.6 | 8.6 | 365 | 3.9 | 67 | 3.0 | 9.0 | 20.0 | 2.0 | 8.0 | 5.5 | 5.4 | 0.2 | 18.8 | 1.2 | 0.55 | 0.04 |  |
| 21 | 8.23 | 7.8 | 12.6 | 578 | 8.0 | 176 | 1.0 | 10.1 | 20.0 | 2.5 | 13.0 | 5.7 | 17.0 | 0.1 | 20.5 | 0.5 | 0.68 | 0.01 |  |
| 22 | 8.14 | 7.0 | 31.6 | 1070 | 29.4 | 95 | 0.5 | 14.7 | 85.0 | 4.6 | 99.0 | 5.1 | 78.4 | 0.2 | 26.3 | 0.9 | 2.41 | 0.14 |  |
| 23 | 8.61 | 3.0 | 45.2 | 562 | 32.5 | 61 | 0.5 | 9.2 | 87.0 | 5.9 | 47.5 | 4.4 | 28.2 | 0.2 | 16.7 | 0.9 | 1.49 | 0.18 |  |
| 24 | 7.61 | 5.8 | 29.5 | 892 | 19.4 | 66 | 1.0 | 6.5 | 112.0 | 11.0 | 222.0 | 2.4 | 3.7 | 0.5 | 22.4 | 0.1 | 1.98 | 0.12 |  |
| 25 | 7.80 | 19.7 | 28.0 | 978 | 16.6 | 248 | 1.0 | 19.1 | 39.0 | 5.4 | 281.0 | 7.7 | 9.1 | 0.3 | 103.0 | 0.2 | 0.74 | 0.06 |  |
